# Acid Stimulation (Sour Taste) Elicits GABA and Serotonin Release from Mouse Taste Cells

**DOI:** 10.1371/journal.pone.0025471

**Published:** 2011-10-20

**Authors:** Yijen A. Huang, Elizabeth Pereira, Stephen D. Roper

**Affiliations:** 1 Department of Physiology and Biophysics, Miller School of Medicine, University of Miami, Miami, Florida, United States of America; 2 Program in Neuroscience, Miller School of Medicine, University of Miami, Miami, Florida, United States of America; Duke University, United States of America

## Abstract

Several transmitter candidates including serotonin (5-HT), ATP, and norepinephrine (NE) have been identified in taste buds. Recently, γ-aminobutyric acid (GABA) as well as the associated synthetic enzymes and receptors have also been identified in taste cells. GABA reduces taste-evoked ATP secretion from Receptor cells and is considered to be an inhibitory transmitter in taste buds. However, to date, the identity of GABAergic taste cells and the specific stimulus for GABA release are not well understood. In the present study, we used genetically-engineered Chinese hamster ovary (CHO) cells stably co-expressing GABA_B_ receptors and Gαqo5 proteins to measure GABA release from isolated taste buds. We recorded robust responses from GABA biosensors when they were positioned against taste buds isolated from mouse circumvallate papillae and the buds were depolarized with KCl or a stimulated with an acid (sour) taste. In contrast, a mixture of sweet and bitter taste stimuli did not trigger GABA release. KCl- or acid-evoked GABA secretion from taste buds was Ca^2+^-dependent; removing Ca^2+^ from the bathing medium eliminated GABA secretion. Finally, we isolated individual taste cells to identify the origin of GABA secretion. GABA was released only from Presynaptic (Type III) cells and not from Receptor (Type II) cells. Previously, we reported that 5-HT released from Presynaptic cells inhibits taste-evoked ATP secretion. Combined with the recent findings that GABA depresses taste-evoked ATP secretion [1], the present results indicate that GABA and 5-HT are inhibitory transmitters in mouse taste buds and both likely play an important role in modulating taste responses.

## Introduction

When mammalian taste buds are stimulated, taste cells release neurotransmitters that excite primary afferent fibers and transmit gustatory signals to the CNS. These transmitters also mediate cell-to-cell interactions within the taste bud that play important roles in shaping the output and generating the taste code for gustatory stimuli. Studies have shown that taste cells synthesize or take up a number of candidate transmitters, including serotonin (5-HT), acetylcholine, neuropeptide Y, norepinephrine (NE), and GABA [1]–[10]. A canonical criterion for identifying neurotransmitters is observing stimulus-evoked release from cells and tissues. To date, only 5-HT, ATP and NE have been so identified [3], [7], [11]–[14]. Specifically, these transmitters have been shown to be secreted by separate classes of taste cells. In response to sour taste stimulation, Presynaptic (Type III) taste cells release 5-HT and NE [3], [7], [14]. Sweet and bitter taste stimuli trigger Receptor (Type II) cells to secrete ATP [12], [13]. In addition to activating gustatory afferent fibers, certain of these transmitters also modulate the function of adjacent taste cells. For example, ATP excites sensory afferents [11] as well as stimulates Presynaptic cells to release 5-HT [12].

GABA (γ-aminobutyric acid) is a major inhibitory transmitter in the mammalian central nervous system. Obata et al. [8] reported that a subset of rat taste bud cells contain GABA as well as GABA transporter subtype 3 (GAT3), and suggested that GABAergic transmission may be involved in the taste sensation. More recently, several laboratories have reported that taste cells express GABA synthetic enzymes, L-glutamic acid decarboxylase subtypes 65 and 67 (GAD65, 67) and GABA receptors [1], [10], [15], [16]. Lastly, exogenous GABA has been shown to increase inwardly rectifying potassium current in rat taste cells [9] and inhibit taste-evoked ATP secretion from Receptor (Type II) cells [1]. In sum, the data strongly implicate GABA as an inhibitory transmitter in mouse taste buds. Nonetheless, despite these findings, GABA release has yet to be unambiguously established in taste buds.

Here we have used genetically-engineered biosensors to measure GABA release from isolated mouse taste buds and/or taste cells and test how GABA acts on taste bud function. The results show unambiguously that GABA is an inhibitory taste transmitter released by Presynaptic (Type III) taste bud cells.

## Materials and Methods

### Animals and Ethics Statement

We used adult mice of either sex including C57BL/6J and GAD67-GFP transgenic [17] mice in this study. A majority of Type III (Presynaptic) cells in GAD67 transgenic mice express green fluorescent protein [18], enabling one to confidently identify Presynaptic cells.

Mice were sacrificed with CO_2_ and followed by cervical dislocation. All procedures were conducted following the guideline of National Institute of Health, as approval by the University of Miami Animal Care and Use Committee (Animal Welfare Assurance Number, A3224-01).

### Isolated taste buds and/or taste cells

Details of how taste buds and cells were isolated are described in Huang et al. [19], [20]. Briefly, we injected an enzyme cocktail containing 1 mg/ml collagenase A (Roche, Indianapolis, IN), 2.5 mg/ml dispase II (Roche, Indianapolis, IN), and 1 mg/ml trypsin inhibitor (Sigma, St. Louis, MO) beneath the epithelium surrounding circumvallate papillae and removed the lingual epithelium. Isolated taste buds were collected in glass micropipettes and transferred to a recording chamber (Warner Inst) with a glass coverslip base. To isolate single taste cells, individual taste buds were triturated in the recording chamber using a glass micropipette. Taste cells were loaded with 5 µM Fura 2 AM following their isolation.

### Biosensor cells

GABA biosensors were obtained from Novartis Institutes for BioMedical Research in Switzerland. GABA biosensors consisted of Chinese hamster ovary (CHO) cells stably expressing heteromeric GABA_B_ (GABA_B_ R1b and GABA_B_ R2) receptors and the G-protein α subunit, Gαqo5 [21], [22]. We also used ATP biosensors [12]. Biosensors were loaded with 5 µM Fura 2 AM as described in Huang et al. [12], [20].

An aliquot of Fura 2-loaded biosensor cells (GABA or ATP) was transferred to the recording chamber containing isolated taste buds and/or isolated taste cells. Immediately after GABA or ATP biosensors had settled to the bottom of the chamber, they were probed with a single application of GABA (10 to 100 nM) or ATP (0.3 to 1 µM). Highly-sensitive biosensors were drawn onto a fire-polished glass micropipette to test transmitter release from taste buds and/or cells. Unless otherwise treated (see next), GABA biosensors also respond to ATP because CHO cells express endogenous P2Y receptors [23]. Thus, prior to conducting experiments, we preincubated GABA biosensors with 500 µM ATP for 30 minutes to desensitize their endogenous purinoceptors for the duration of the testing. This procedure has been previously described and documented [7], [12].

Controls to establish the sensitivity and selectivity of GABA biosensors were similar to those described for 5-HT, NE, and ATP biosensors in Huang et al. [3], [7], [12], [19], [20] and Dvoryanchikov, Huang et al. [1]. Namely, we verified that GABA biosensors alone do not respond to bath-applied KCl (50 mM) or to the taste stimuli used in this study (see below). CGP55845 (10 µM), a GABA_B_ receptor antagonist, was used to verify that responses in biosensor cells were generated by GABA receptors. Lastly, we verified that biosensors were not affected by the pharmacological agents used in this study, apart from CGP55845.

### Stimuli and solutions

All stimuli and pharmacological agents were made in Tyrode's buffer (in mM; 140 NaCl, 5 KCl, 2 CaCl_2_, 1 MgCl_2_, 10 HEPES, 10 glucose, 10 Na-pyruvate, 5 NaHCO_3_, pH 7.2, 310–320 Osm) and applied at pH 7.2 except for acetic acid. Acetic acid was applied at pH 5.0. For Ca^2+^-free Tyrode's solution, MgCl_2_ was substituted for CaCl_2_. Bicuculline and WAY100635 were obtained from Sigma (Saint Louis, MO) and CGP55845 was obtained from Tocris (Park Ellisville, MO).

Isolated taste buds and/or cells were stimulated by bath-perfusion of KCl (50 mM substituted equimolar for NaCl), a sweet/bitter taste mix (cycloheximide, 10 µM; saccharin, 2 mM; SC45647, 0.1 mM; denatonium, 1 mM), or acetic acid (10 mM, pH 5.0). We bath-applied stimuli for 30 seconds followed by return to Tyrode's buffer for 3∼5 minutes between trials, as described in Huang et al. [19]. We have found that this perfusion paradigm allows the stimuli to mix thoroughly with the recording chamber, reach a final concentration in the bath followed by a complete washout, and produces reliable and consistent responses that are stable over repeated trials.

### Ca^2+^ imaging

For Fura 2-loaded biosensor and isolated taste cells, images were recorded at 40× with excitation at 340 nm followed by 380 nm (*e.g.* [12]). Images were processed with Indec Workbench v5 software. The F340/F380 ratio was converted to approximate [Ca^2+^]_i_ as described by Grynkiewicz et al. [24] and using a Fura 2 Calcium Imaging calibration Kit (Invitrogen, USA). Average baseline [Ca^2+^] in these experiments was 181±15 nM (N = 19 cells), in good correspondence with values reported previously [20], [25].

Our criteria for accepting Ca^2+^ responses for analysis are described in Huang et al. [19]. In brief, responses were quantified as peak response minus baseline [Ca^2+^] (i.e. Δ[Ca^2+^]). We accepted Ca^2+^ responses only if they could be elicited repetitively in the same cell by the same stimulus, and evoked responses were at least 2× baseline [Ca^2+^] fluctuation. All experiments were performed at room temperature (25°C).

## Results

**Figure 1:**
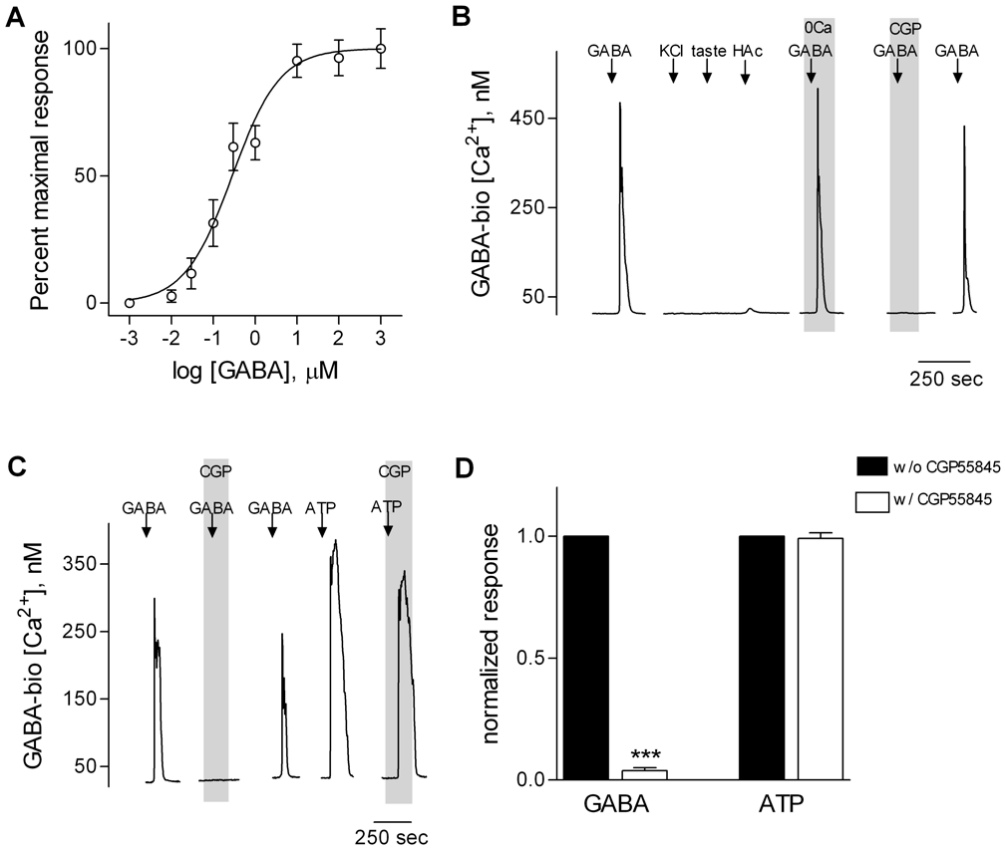
CHO cells stably expressing GABA_B_ receptors are sensitive and reliable GABA biosensors. Biosensors were loaded with Fura 2 and Ca^2+^ mobilization was measured in response to bath-applied GABA. **A,** Concentration-response relationships for GABA (symbols show mean ± s.e.m., N = 14 cells). **B,** Representative traces from a GABA biosensor showing that the biosensors do not respond to KCl depolarization (↓, KCl) or taste stimulation with a sweet/bitter mixture (↓, taste), and only minimally to acetic acid (↓, HAc). GABA-evoked Ca^2+^ responses (↓, GABA) were unaffected by removing extracellular Ca^2+^ (“0 Ca”, throughout the shaded area). Lastly, CGP55845 (“CGP”, 10 µM, present throughout the shaded area) reversibly blocked GABA-elicited biosensor responses. GABA, 100 nM; KCl, 50 mM. **C,** Representative traces from a GABA biosensor showing that biosensors respond to either GABA (100 nM) or ATP (10 µM), but CGP55845 (10 µM, present throughout the shaded area) only blocks responses elicited by GABA. **D,** Summary of data as shown in C. N = 6; ***, *p*<0.001, Student *t*-test.

We tested whether gustatory stimuli excite taste buds to release GABA and if so, which type(s) of taste cells were responsible for the release. We used CHO cells stably transfected with GABA_B_ receptors as GABA biosensors. Biosensors were loaded with the Ca^2+^-sensitive dye Fura 2 and tested with bath-applied GABA in the absence of taste buds and taste cells. Threshold activation was ∼30 nM GABA on average, although individual cells responded to concentrations as low as 10 nM. Concentration-response relations for biosensors indicated that EC_50_ for GABA was 258 nM (Fig. 1A), consistent with the FLIPR (Fluorescent Imaging Plate Reader) data from the provider of this cell clone (see Materials and Methods, unpublished data). Biosensor responses to bath-applied GABA were reversibly blocked by 10 µM CGP55845, a selective GABA_B_ receptor antagonist (Figs. 1B, 1C). GABA biosensors did not significantly respond to depolarization with bath-applied KCl (50 mM, substituted for NaCl), to a mixture of bitter and sweet taste compounds (see Methods), or to acetic acid (HAc, 10 mM, pH 5.0) (Fig. 1B). GABA biosensors showed robust Ca^2+^ mobilization in response to GABA when Ca^2+^ in the medium was replaced with Mg^2+^ (0 Ca, Fig. 1B), consistent with coupling of GABA_B_ receptors to intracellular Ca^2+^ store release [21], [22]. If biosensors were not desensitized prior to the experiment (see Materials and Methods), they responded to ATP (via endogenous purinoceptors) as well as to GABA. Thus we used CGP55845 to verify that biosensor responses were generated by activating GABA receptors, not endogenous ATP receptors (CGP, Figs. 1C, 1D). (Responses to ATP were unaffected by 10 µM CGP55845, but those to GABA were blocked). In short, GABA biosensors are highly sensitive, reliable, and specific detectors for GABA.

**Figure 2:**
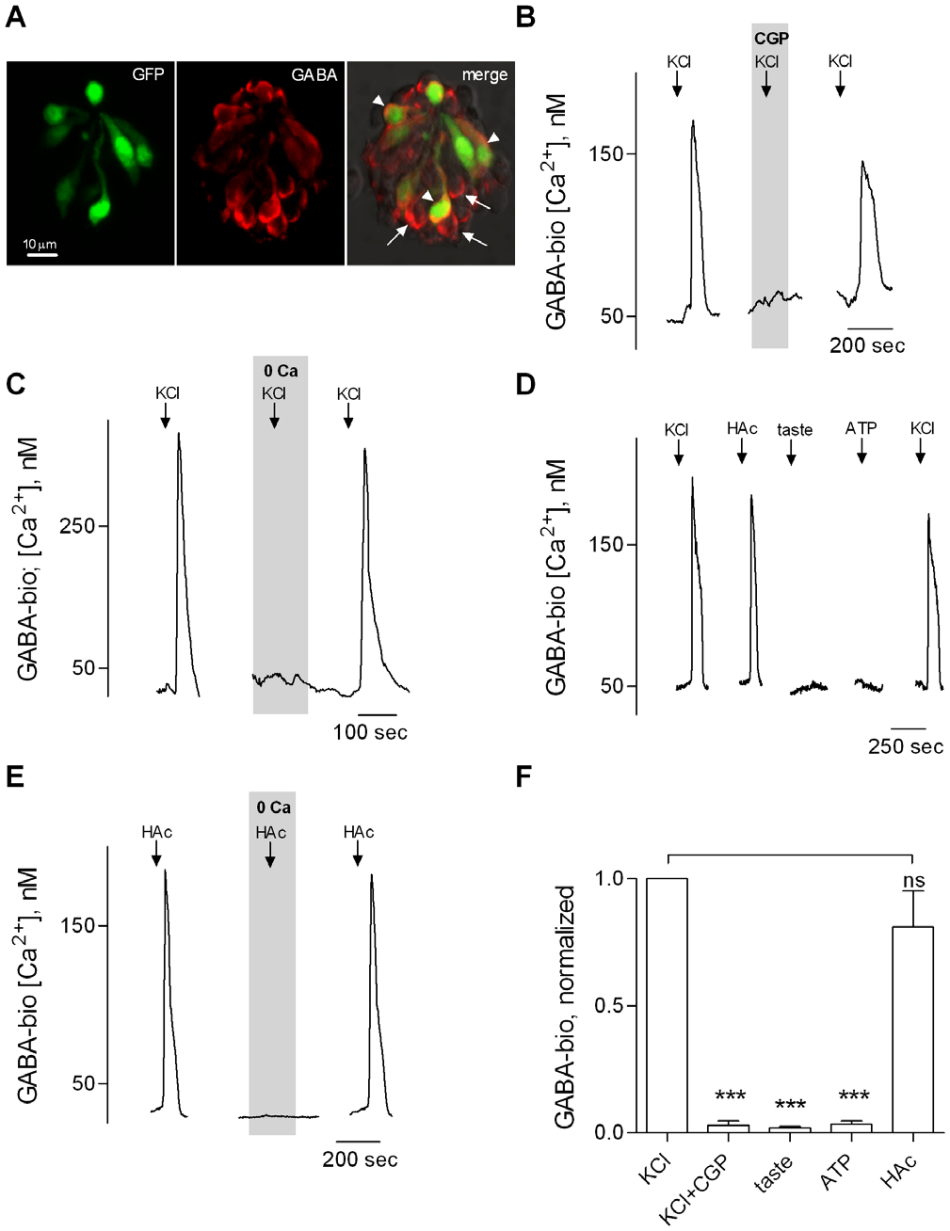
Stimulating taste buds evokes GABA release. **A,** GABA immunostaining of an isolated, fixed taste bud from a GAD67-GFP mouse. Many GABA immunopositive taste cells express GFP (arrowheads), but some do not (arrows). This latter category may be Type I cells. **B,** Traces show robust responses from a biosensor positioned against a taste bud when the taste bud was depolarized by 50 mM KCl (↓, KCl). The GABA receptor antagonist CGP55845 (“CGP”, 10 µM, present throughout the shaded area) reversibly abolished KCl-evoked biosensor responses. Withdrawing the biosensor from the taste bud eliminated all responses to KCl (as in Fig. 1, data not shown). **C,** Another taste bud. KCl-evoked biosensor responses (↓, KCl) were abolished when Ca^2+^ was replaced with Mg^2+^ in the bathing medium (“0Ca”, throughout shaded area). **D,** KCl (50 mM) depolarization and sour taste stimulation (10 mM acetic acid, pH 5.0, HAc) triggered GABA release from another taste bud. In contrast, a sweet/bitter taste mix (taste) or ATP (1 µM), did not stimulate GABA release. **E,** Acetic acid-evoked GABA release (↓, HAc) was eliminated when extracellular Ca^2+^ was removed (“0Ca”, throughout shaded area). **F,** Summary of data. Stimulating taste buds with 50 mM KCl (N = 7) or acetic acid (HAc, N = 6) but not a sweet/bitter taste mix (taste, N = 6) or ATP (N = 5) evokes GABA release. ns, not significant. ***, *p*<0.001, Student *t*-test.

Subsequently, we isolated taste buds from vallate papillae. GABAergic taste cells could readily be observed in immunostained isolated taste buds. In taste buds isolated from GAD67-GFP mice, many GABA immunopositive taste cells expressed green fluorescent protein (GFP), consistent with previous reports that these are GABAergic Presynaptic (Type III) taste cells (Fig. 2A) [1], [18]. In addition, some GABA immunopositive cells lacked GFP, suggesting these cells were Type I cells [1]. These results verify that GABAergic cells were present and that their GABA content was maintained throughout the isolation procedure.

To test whether isolated taste buds are capable of releasing GABA, we depolarized mouse taste buds with 50 mM KCl, an effective stimulus for GABAergic Presynaptic (Type III) cells. GABA biosensors abutted against individual taste buds showed a robust Ca^2+^ signal when the taste buds were depolarized with KCl (Fig. 2B). (As stated above, biosensors in the absence of taste buds showed no responses to KCl). CGP55845 reversibly blocked KCl-elicited biosensor responses, confirming the identity of the released amino acid. These findings indicate that taste cells release GABA when they are depolarized. KCl-evoked GABA release required Ca^2+^ in the bathing medium (Fig. 2C).

Next, we tested whether isolated taste buds release GABA during gustatory stimulation. Unexpectedly, we found that a mixture of sweet and bitter tastants failed to trigger taste buds to release GABA (Fig. 2D). Previously, we had shown that taste stimulation evokes secretion of multiple transmitters, including ATP, serotonin (5-HT), and norepinephrine (NE) [3], [7], [12]. Moreover, ATP itself stimulates 5-HT and NE secretion by stimulating Presynaptic cells [12]. Thus, we tested whether bath-applied ATP stimulated taste buds to release GABA. Figure 2D shows that ATP also failed to evoke GABA release from taste buds. Importantly, however, sour taste (acetic acid) stimulation did trigger GABA secretion (Fig. 2D, 2E). As also found for depolarization-evoked GABA secretion, acid-evoked GABA release was eliminated when extracellular Ca^2+^ was substituted with Mg^2+^ (Fig. 2E). Parenthetically, sour taste is an effective stimulus for Presynaptic (Type III) cells [14], [18].

In our previous study using 5-HT biosensors and KCl depolarization, we found that biosensors detected 5-HT release in 39% of taste buds (213/540) [7]. This incidence of detection reflects the requirement that biosensors must be appropriately positioned relative to the location of the 5-HT-secreting cell(s) within the taste bud and is certainly an underestimate of the actual incidence of stimulus-evoked secretion. In the present study, biosensors detected KCl-stimulated GABA release in 32% of the taste buds (7/22). The similarity of these findings suggests that taste buds may co-release GABA with 5-HT, as occurs with NE and 5-HT [7]. This possibility, though intriguing, awaits further study.

**Figure 3:**
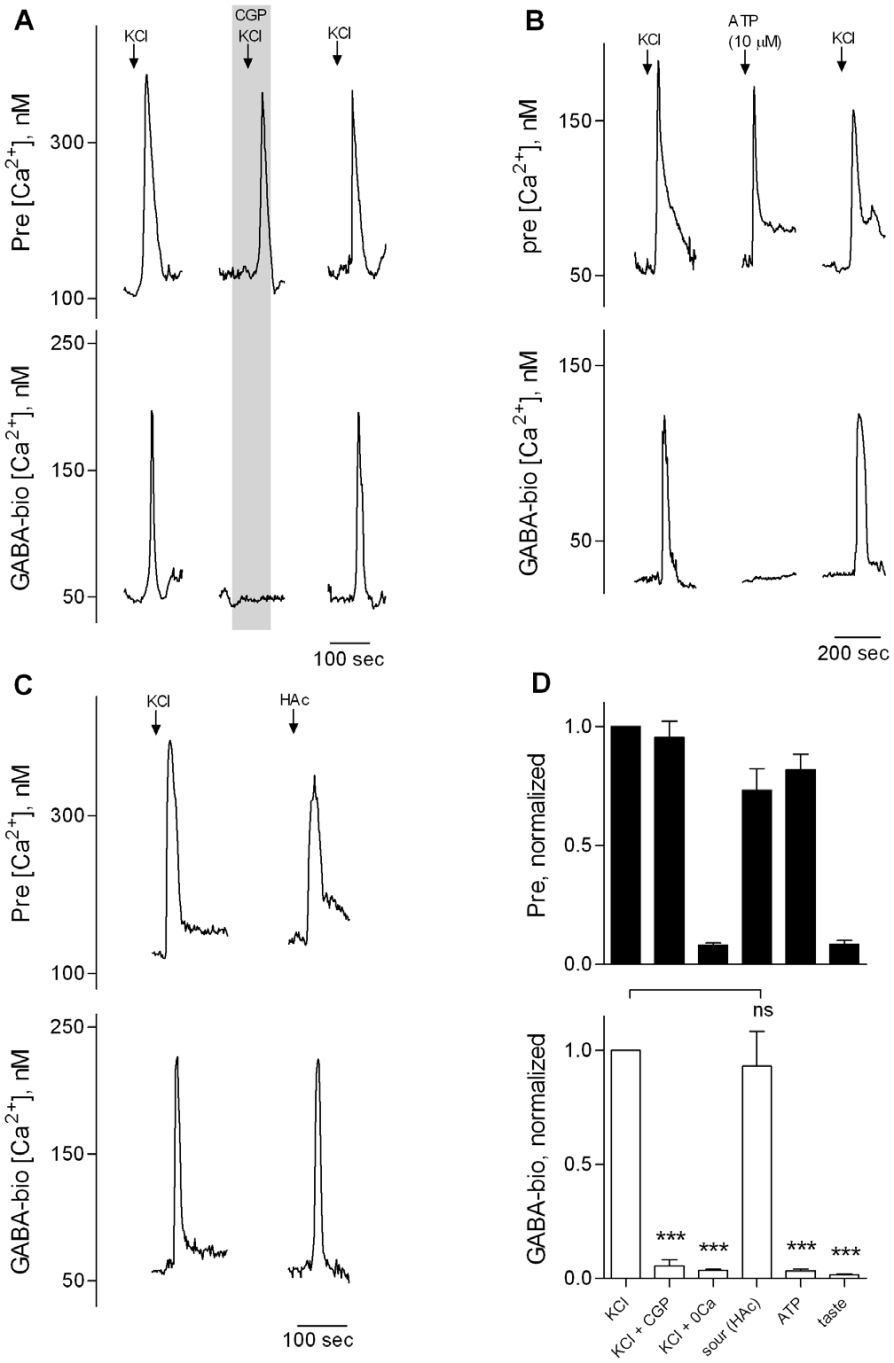
Stimulating Presynaptic (Type III) taste cells evokes GABA release. GAD-GFP mice were used to isolate and identify Presynaptic taste cells. **A,** Traces show concurrent Ca^2+^ recordings from an isolated Presynaptic cell (upper traces, Pre) and an apposed GABA biosensor (lower traces, GABA-bio). Stimulating the Presynaptic cell with 50 mM KCl (↓, KCl) evoked a transient Ca^2+^ response and the biosensor reported that GABA was released (lower trace). CGP55845 (10 µM, “CGP”, present throughout shaded area) reversibly blocked the biosensor responses without affecting Presynaptic cell responses. **B,** From another experiment, KCl and ATP (10 µM) evoked Ca^2+^ responses in a Presynaptic cell (upper traces) but only KCl depolarization triggered GABA release (lower traces). **C,** Another experiment, 50 mM KCl and 10 mM acetic acid (HAc, pH 5.0) evoked a transient Ca^2+^ elevation in the Presynaptic cell (upper traces) and triggered GABA release (lower traces). **D,** Summary of data from identified Presynaptic cells. KCl depolarization (N = 4) and sour tastant (HAc, N = 4) stimulate Presynaptic cells to release GABA. CGP55845 (CGP) inhibits KCl-evoked biosensor responses. Neither tastants (sweet/bitter taste mixture, N = 3) nor ATP (N = 4) stimulates Presynaptic cells to release GABA. ns, not significant. ***, *p*<0.001, , Student *t*-test.

Taste cells synthesize and store GABA (rat, [9]; mouse, [1], [10]). Specifically, Presynaptic (Type III), but not Receptor (Type II) cells express GABA biosynthetic enzymes and are immunopositive for GABA (Fig. 2A) [1]. Thus, we investigated whether Presynaptic cells release GABA. Using transgenic mice expressing GFP in GAD67 (Presynaptic Type III) cells [12], [15], [18], we isolated and identified individual Presynaptic cells from circumvallate taste buds. We loaded taste cells with Fura 2, positioned a GABA biosensor cell against a Presynaptic cell, and tested whether KCl-depolarization or acid stimulation triggered GABA secretion. Consistent with our hypothesis, both KCl and acid stimulation evoked GABA release from isolated Presynaptic cells (Fig. 3). CGP55845 reversibly abolished these biosensor responses, verifying that Ca^2+^ responses in those biosensors truly reflected GABA release (Fig. 3A).

Previously, we demonstrated that ATP stimulates isolated Presynaptic cells to secrete 5-HT in a Ca^2+^-dependent manner, indicative of P2Y-mediated intracellular Ca^2+^ mobilization and not Ca^2+^ influx [12]. Here, as with taste buds (above), we tested whether ATP stimulated isolated Presynaptic cells to release GABA. Interestingly, ATP (up to 10 µM) generated robust Ca^2+^ mobilization in Presynaptic cells but did not trigger GABA release (Fig. 3B). This result was consistent with the failure of taste stimulation of taste buds, which evokes ATP secretion from Receptor cells, to trigger GABA secretion (Fig. 2D). In marked contrast, acetic acid stimulated Ca^2+^ influx in Presynaptic cells and triggered GABA release (Fig. 3C). Collectively, these data show that KCl depolarization or acid stimulation evokes Ca^2+^-dependent GABA secretion from taste buds, that the source of the GABA is the sour-sensing Presynaptic cells, and that Presynaptic cells can secrete different transmitters (5-HT, NE, GABA) under different mechanisms (5-HT and NE upon Ca^2+^ influx or intracellular Ca^2+^ mobilization; GABA only upon Ca^2+^ influx) [3], [7], [12].

**Figure 4:**
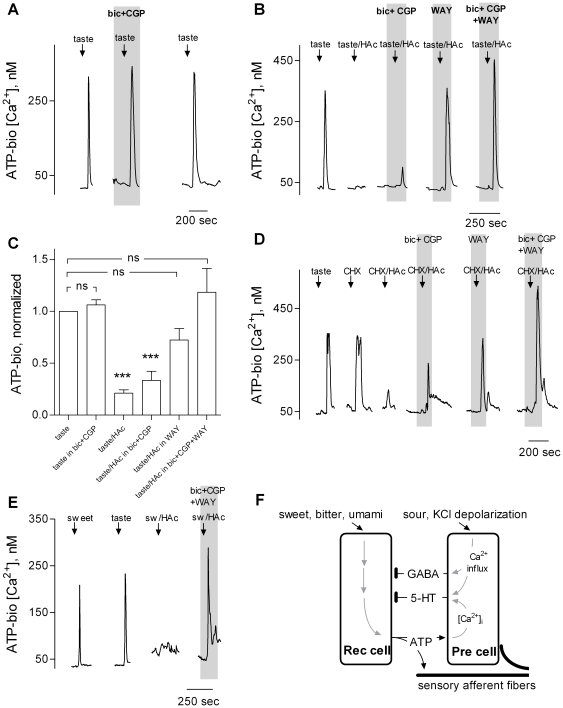
Sour taste initiates GABAergic and serotonergic inhibition in taste buds. ATP biosensors were positioned against taste buds to measure taste-evoked transmitter secretion [12]. **A,** Traces show ATP biosensor responses. Stimulating the taste bud with a sweet/bitter taste mix (↓, taste) triggered responses in the biosensor, i.e. ATP secretion. Taste-evoked ATP secretion was unaffected by blocking GABA receptors with bicuculline and CGP55845, GABA_A_ and GABA_B_ receptor antagonists, respectively (“bic+CGP”, present throughout the shaded area). **B,** In another experiment, ATP secretion stimulated by sweet/bitter tastants (as in A) was strikingly diminished when a sour tastant (HAc, acetic acid 10 mM, pH 5.0) was added to the stimulus mix (↓, taste/HAc). The reduction of ATP secretion was partially restored by blocking GABA receptors (“bic+CGP”, present throughout shaded area). In this experiment, the acid-evoked inhibition was completely rescued by blocking 5-HT receptors with WAY100635, a 5-HT_1A_ receptor antagonist, (“WAY”, present throughout shaded area). Blocking GABA and 5-HT receptors together resulted in an even larger taste-evoked ATP secretion from this taste bud (“bic+CGP+WAY”, present throughout shaded area). **C,** Summary of data from several experiments. Taste stimulation with a sweet/bitter mix causes taste buds to secrete ATP (N = 28). However, taste mix in the presence of an acid stimulus results in decreased transmitter (ATP) secretion (N = 23). Blocking 5-HT_1A_ receptors (N = 4) or GABA_A_/GABA_B_ receptors separately partially restores the taste-evoked ATP secretion (N = 16). Blocking GABA and 5-HT receptors together fully restores taste-evoked ATP secretion (N = 8). ns, not significant; ***, p<0.001, Student *t*-test. **D,** In a separate experiment, taste mix or a bitter taste compound alone (10 µM cycloheximide, CHX), elicits transmitter secretion. As in B, ATP secretion was significantly diminished when a sour tastant was added to the bitter stimulus (↓, CHX/HAc). Partial restoration of ATP secretion was observed by either adding GABA receptors antagonists (“bic+CGP”, present throughout shaded area) or a 5-HT_1A_ receptor antagonist (“WAY”, present throughout shaded area) in the bath medium. As expected, in this experiment, blocking GABA and 5-HT receptors together resulted in an even larger bitter taste-evoked ATP secretion from this taste bud (“bic+CGP+WAY”, present throughout shaded area). **E,** A sweet taste compound alone (0.1 mM SC45647, SW) and taste mix elicit transmitter secretion; the combination of sweet taste and acid stimulus (sw/HAc) resulted in little, if any, ATP release. As in D, blocking GABA and 5-HT receptors together resulted in an even larger sweet taste-evoked ATP secretion from this taste bud (“bic+CGP+WAY”, present throughout shaded area). **F,** Schematic diagram illustrating the findings. Sweet, bitter (and umami) tastes stimulate Receptor cells (Rec) to secrete ATP. KCl depolarization or sour taste stimulation triggers Ca^2+^ influx in Presynaptic cells (Pre) and evokes GABA and 5-HT secretion. ATP triggers intracellular Ca^2+^ mobilization in and 5-HT release from Presynaptic cells. Parenthetically, ATP acts on sensory afferent fibers as well.

Finally, we examined the role GABA plays during taste stimulation in whole taste buds where cell-to-cell communication remained intact. Our hypothesis is that GABA, like serotonin, mediates inhibitory interactions within the taste bud during gustatory stimulation. We measured gustatory-evoked ATP secretion from intact taste buds using ATP biosensors [12] and tested whether GABAergic transmission altered this release. Figure 4A shows that blocking GABA_A_ and GABA_B_ receptors using CGP55845 and bicuculline, respectively, did not alter ATP secretion evoked by a mixture of sweet and bitter compounds. This suggests that GABAergic mechanisms are not involved in sweet/bitter taste, *per se*. In contrast, sweet/bitter-evoked ATP secretion was significantly reduced when a sour tastant (HAc, 10 mM acetic acid, pH 5.0) was added to the taste mix (arrows, taste/HAc, in Fig. 4B), consistent with acid-evoked GABAergic inhibition. Repeating sweet/bitter plus acid stimulation in the presence of CPG55845 and bicuculline partially rescued taste-evoked ATP secretion, also consistent with acid-evoked GABA secretion having contributed to the depressed ATP output during sour stimulation. That ATP release was only partially rescued by blocking GABAergic transmission suggested that an additional inhibitory mechanism might be recruited by acid stimulation. Acid taste stimulates Presynaptic cells to release GABA (Fig. 3) and 5-HT [14]. Furthermore, 5-HT also inhibits Receptor cells from secreting ATP [19]. Thus, we tested whether blocking serotonergic mechanisms in combination with blocking GABAergic inhibition would fully restore ATP secretion. Figures 4B and 4C show that sweet/bitter taste-evoked ATP secretion was completely rescued from sour taste inhibition when GABA receptors were blocked by CPG55845 and bicuculline, and WAY100635, a selective 5-HT_1A_ receptor antagonist was present [19].

Parenthetically, taste-evoked ATP release in the presence of the combined serotonergic and GABAergic inhibitors was sometimes even greater than control ATP release (i.e. in the absence of inhibitors) (see Fig. 4B,D,E). One might expect this increase in taste-evoked ATP secretion over control because in the presence of WAY100635, ongoing negative feedback from endogenous serotonergic mechanisms (i.e., 5-HT released from Presynaptic cells during “control” trials) is blocked [19]. However, the increased release of ATP in the presence of the combined inhibitors was, on average, not statistically significant (Fig. 4C).

In brief, our findings show that sour taste activates a combination of GABAergic and serotonergic inhibitory pathways in taste buds, which, together, reduce transmitter output evoked by sweet/bitter taste stimulation. As a final test, we repeated the same procedures as above but using only bitter (cycloheximide, CHX, Fig. 4D) or sweet (Fig. 4E) stimuli alone instead of sweet/bitter taste mixtures. Our data showed that either bitter or sweet tastants elicited ATP secretion, and that these responses, like those to the sweet/bitter mixture (Fig. 4B), were inhibited by sour-evoked GABA/5-HT release. Figure 4F presents a schematic diagram of the tandem GABAergic and serotonergic inhibitory pathways.

## Discussion

The present findings indicate that sour taste stimulation and KCl depolarization cause Presynaptic (Type III) taste bud cells to secrete GABA and that GABA inhibits transmitter secretion from taste Receptor (Type II) cells. Presynaptic taste cells also secrete serotonin (5-HT). GABA and 5-HT combine to inhibit ATP secretion when taste buds are stimulated with sweet and bitter tastants. How this interplay of GABAergic and serotonergic mechanisms in taste buds shapes taste responses and taste behaviour in the intact animal remains to be investigated.

Presynaptic (Type III) cells in circumvallate taste buds of mice express the GABA synthetic enzyme GAD67 and Type I taste bud cells express GAD65 [10], [15], [18]. In the present study, we clearly showed that Presynaptic cells release GABA. We have no information on whether Type I cells might also secrete GABA and if so, what might be the effective stimulus for such release. It may be important that in intact taste buds, sweet and bitter taste stimuli, which excite Receptor (Type II) cells, did not trigger GABA release but sour taste stimulation, which excites Presynaptic (Type III) cells, did. We did not investigate salt taste (Na^+^), which may be an effective stimulus for Type I cells [26], [27]. It remains to be determined whether Type I cells contribute to GABAergic mechanisms in taste buds.

Presynaptic cells themselves express GABA receptors [1], [10] and thus one might speculate that GABA released from these cells might exert autocrine feedback. We have tested whether GABA acts on Presynaptic cells, and specifically whether GABA affects transmitter release from these cells. We found that 5-HT release from Presynaptic cells, evoked by KCl depolarization- or by ATP, was unaltered by the GABA agonists, muscimol or baclofen [1]. An autocrine action of GABA on Presynaptic cells remains an intriguing question, but we have no evidence for it.

Previously, we reported that intracellular Ca^2+^ release as well as Ca^2+^ influx elicits 5-HT release from Presynaptic taste cells [12], [19]. Our present findings indicate that only Ca^2+^ influx evokes GABA release from these same cells. This suggests that Presynaptic cells release different neurotransmitters by using different Ca^2+^ sources. Store-operated Ca^2+^ channels (SOCs) may be one such Ca^2+^ source for transmitter secretion from taste buds [28]. However, eliminating Ca^2+^ in the bathing medium does not eliminate taste-evoked 5-HT secretion from taste buds [3], and ATP, which triggers SOCs in taste buds [29], does not evoke GABA release (present results). In combination, those results suggest that SOCs might not be involved in transmitter release mechanisms, at least for 5-HT and GABA.

The strict requirement for Ca^2+^ influx for GABA secretion may indicate that there is a restricted location of the Ca^2+^-activated release sites close to the plasma membrane that leads to exocytosis of synaptic vesicles from Presynaptic cells [30]. Recently, Deshpande et al. [31] reported that bitter compounds cause relaxation of isolated airway smooth muscle. The relaxation is associated with a Ca^2+^ response localized to the cell membrane which opens large-conductance, Ca^2+^-activated K^+^ channels, leading to membrane hyperpolarization. This is distinct from histamine-induced increases in intracellular Ca^2+^ throughout these same cells which leads to an opposite end result―depolarization and contraction. Whether there are comparable distinctive Ca^2+^-activated mechanisms in Presynaptic cells for GABA release *versus* 5-HT secretion remains an intriguing possibility to be tested.
